# Development of CBCT-Guided Online Adaptive Radiotherapy

**DOI:** 10.3390/cancers18172755

**Published:** 2026-08-25

**Authors:** Katsuyuki Shirai, Noriko Kishi, Hideaki Hirashima, Masashi Endo, Rihito Aizawa, Masanori Takaki, Tadamasa Yoshitake, Tomohiro Harasawa, Soichiro Ito, Yoosuk Kang, Takashi Mizowaki

**Affiliations:** 1 Department of Radiology, Jichi Medical University Hospital, Shimotsuke 329-0498, Japan; r1707em@jichi.ac.jp (M.E.); yoosuk@jichi.ac.jp (Y.K.); 2 Department of Radiation Oncology and Image-Applied Therapy, Graduate School of Medicine, Kyoto University, Kyoto 606-8507, Japan; kishin@kuhp.kyoto-u.ac.jp (N.K.); hhideaki@kuhp.kyoto-u.ac.jp (H.H.); ra0920@kuhp.kyoto-u.ac.jp (R.A.); mizo@kuhp.kyoto-u.ac.jp (T.M.); 3 Department of Clinical Radiology, Graduate School of Medical Sciences, Kyushu University, 3-1-1, Maidashi Higashi-Ku, Fukuoka 812-8582, Japan; takaki.masanori.328@m.kyushu-u.ac.jp (M.T.); yoshitake.tadamasa.386@m.kyushu-u.ac.jp (T.Y.); 4 Department of Radiology, Graduate School of Medical and Dental Sciences, Kagoshima University, Kagoshima 890-8544, Japan; t.harasawa1992@gmail.com (T.H.); tarotaro@m3.kufm.kagoshima-u.ac.jp (S.I.)

**Keywords:** online adaptive radiotherapy, CBCT, HyperSight, SBRT

## Abstract

**Background:** Online adaptive radiotherapy (oART) can change the dose distribution briefly to match the shape of the targets and organs at risk. Cone-beam computed tomography (CBCT)-guided oART has been introduced in clinical practice and is expected to improve clinical outcomes. This technology allows treatment as an extension of conventional CT-based image-guided radiotherapy technology and has the advantage of relatively short treatment times. However, the clinical and dosimetric benefits of oART have not yet been adequately reported. **Methods**: We systematically reviewed previous studies evaluating CBCT-guided oART in patients with malignancies. Case reports, conference abstracts, letters, editorials, and reviews were excluded. PubMed was searched from January 2019 to 22 November 2025, supplemented by hand searching. Study design, cancer site, workflow, treatment time, dosimetric outcomes, feasibility, and clinical outcomes were extracted. Owing to clinical and methodological heterogeneity, findings were synthesized narratively. **Results:** A total of 127 studies were included. Across most studies, adapted plans improved target coverage and reduced or maintained doses to organs at risk compared with scheduled plans. This review provides an overview of the workflows and latest developments in CBCT-guided oART. Furthermore, the clinical and dosimetric benefits at each site, including the chest, abdomen, pelvis, head, and neck, are shown. **Conclusions:** The development of oART is expected to lead to the introduction of personalized medicine and improve the clinical outcomes of patients with various cancers. Further research and clinical trials are warranted to establish CBCT-guided oART. This review received no external funding and was registered in PROSPERO (CRD420251237632).

## Simple Summary

Online adaptive radiotherapy (oART) is a highly effective treatment technique when the targets and organs at risk change in size and shape. This method is expected to offer precise treatment and reduce the planning target volume margin. Therefore, it improves dose distribution and clinical outcomes without severe adverse effects. Cone-beam computed tomography-guided oART was introduced in 2020 and provides rapid treatment compared with magnetic resonance imaging-guided ART. This review demonstrates the effectiveness of oART for representative cancer types. We hope that this manuscript will be helpful for introducing oART to various institutions.

## 1. Introduction

Radiotherapy (RT) is widely used as a curative or palliative treatment for various tumors. However, the daily movement of tumors and surrounding organs within the body is a major problem that can reduce the success rate of radiation therapy. Image-guided radiotherapy (IGRT) is a technique that corrects the tumor axis according to the daily displacement of the tumor by using cone-beam computed tomography (CBCT) images acquired immediately before treatment, thus contributing to the development of high-precision RT. However, when the target changes in size and shape, it is difficult to modify the treatment plan by using IGRT. Online adaptive RT (oART) allows for the immediate adjustment of dose distribution to match the shape of the target or organs at risk (OARs) by using images taken immediately before treatment. oART is highly effective during RT, particularly at treatment sites where the size and shape of the target and OARs tend to change daily. This technology is irreplaceable and is expected to improve dose distribution and treatment outcomes.

The classification of oART has been reported by Bertholet et al. [1]. There are two types of oART: online plan library, in which plans are prepared in advance and the best plan is chosen one on the day of treatment, and daily online replanning, in which the plan is modified using imaging on the day of treatment. This review discusses daily online replanning as oART. oART was first commercialized with the introduction of magnetic resonance imaging (MRI)-guided systems. MR-guided oART takes advantage of the superior soft-tissue contrast of MRI, allowing detailed assessment of daily changes in the position and shape of the tumor and OARs [2]. This is particularly advantageous for oART in regions such as the abdomen and pelvis, where soft-tissue anatomy can change substantially between treatment fractions. Another important advantage of MR-guided oART is that it does not involve ionizing radiation, allowing continuous real-time monitoring during treatment. However, it also entails challenges such as high costs, long imaging times, safety considerations associated with MRI, and the acquisition of electron density information required for treatment planning. Since 2020, CBCT-guided oART using the ETHOS^TM^ therapy system (Varian Medical Systems, Palo Alto, CA, USA) has been introduced into clinical practice and is expected to enable treatment as an extension of conventional CT-based IGRT technology, thus offering the advantage of relatively short treatment times [3]. Given that prolonged treatment time can exacerbate intrafractional motion, it is important to create a rapid treatment plan by using CBCT and to immediately decide on a treatment plan. The latest ETHOS HyperSight^TM^ CBCT imaging system enables high-speed and high-quality CBCT imaging, thereby improving the accuracy of contour visualization and treatment planning [4]. These are essential for performing precise oART. To briefly summarize the differences between these technologies, MR-guided oART is particularly advantageous when accurate visualization of soft-tissue changes is essential, whereas CBCT-guided oART offers faster and more efficient image-guided adaptation.

In recent years, an increasing number of reports have indicated that CBCT-guided oART is a promising treatment for several types of tumors and is capable of reducing planned target volume (PTV) margins without compromising local control. However, the methods and techniques for oART at each disease site have not yet been fully established. In this article, we review the ETHOS workflow and high-quality CBCT images. Furthermore, we summarize the dosimetric and clinical benefits for each site, including the chest, abdomen, pelvis, head, and neck. A systematic review was performed to collect and summarize the existing research.

## 2. Systematic Reviews

### 2.1. Protocol and Registration

This systematic review was conducted according to the Preferred Reporting Items for Systematic Reviews and Meta-Analyses (PRISMA) 2020 guidelines (Table S1) [5]. The review protocol was prospectively registered in the International Prospective Register of Systematic Reviews (registration number: CRD420251237632). A separate review protocol was not prepared. The PROSPERO record was updated after its initial publication. Version 1.0 was published on 26 December 2025, version 1.1 on 1 January 2026, version 1.2 on 12 May 2026, and version 2.0 on 28 May 2026. These updates reflected clarifications and additions to the registered information and did not materially alter the review objectives, eligibility criteria, literature-search methods, or data-synthesis approach.

### 2.2. Eligibility Criteria

Studies were considered eligible if they met the following inclusion criteria: original articles evaluating online CBCT-guided oART in any cancer type and studies written in English and published in peer-reviewed journals. The exclusion criteria were as follows: case reports, conference abstracts, letters, editorials, or review articles, as well as articles not related to CBCT-guided oART.

A systematic literature search was conducted on PubMed on 22 November 2025, which was restricted to articles published from 2019. The search terms included: (“adaptive radiotherapy”[tiab] OR “adaptive radiation therapy”[tiab]) AND (hasabstract[text] AND (english[lang] OR “english abstract”[Publication Type])) AND (“2019/01/01”[Date—Publication]: “3000”[Date—Publication]) NOT (“Case Reports”[Publication Type] OR “case report”[Title] OR “case reports”[Title] OR MRI[tiab] OR “magnetic resonance”[tiab] OR MRgRT[tiab] OR “MR-guided”[tiab] OR “MR guided”[tiab]).

Additionally, references of relevant studies were manually screened to identify further eligible articles.

### 2.3. Study Selection

After removing duplicates, two reviewers independently screened the titles and abstracts for eligibility, followed by a full-text assessment of potentially relevant studies.

Discrepancies were resolved by discussion or consultation with a third reviewer.

**Figure 1:**
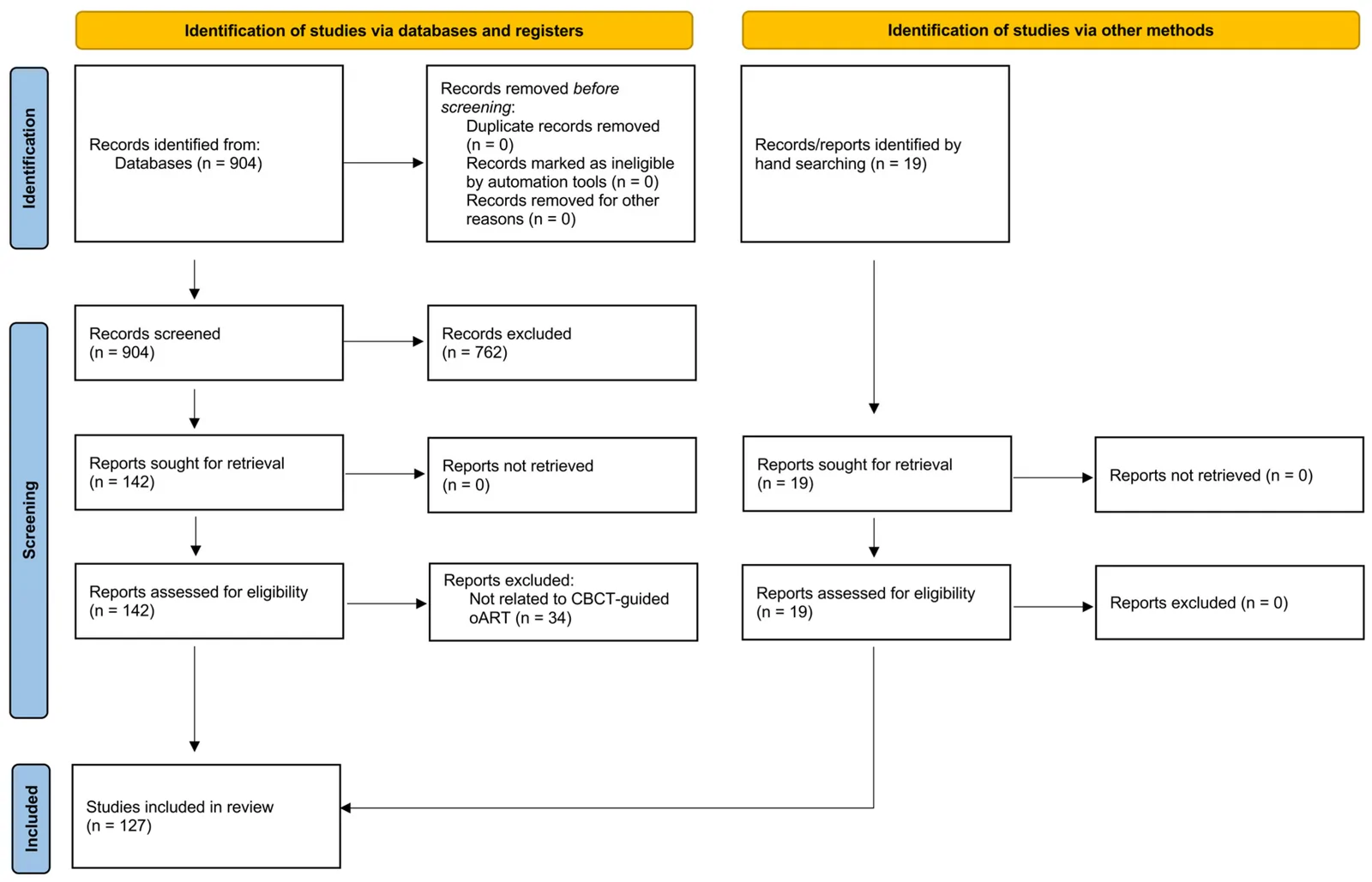
PRISMA 2020 flow diagram for new systematic reviews, including searches of databases and citation searching.

The study selection process is summarized in a PRISMA 2020 flow diagram (Figure 1).

### 2.4. Data Collection Process

The following data were extracted: study characteristics, such as author, year, country, study design, and cancer site; endpoints (feasibility, clinical outcomes, dosimetric outcomes, and treatment time); and results.

### 2.5. Data Synthesis and Statistical Analysis

Owing to the heterogeneity in cancer sites, comparator plans, and outcome measures, a quantitative meta-analysis was not feasible. Instead, a narrative synthesis was conducted to summarize the effects of CBCT-guided oART across studies. The findings were organized according to cancer site, workflow, CT simulation (CT-sim)-free methods, and reported dosimetric outcomes. Emphasis was placed on the clinical outcomes, patterns of improvement in target coverage, sparing of OARs, and study-level conclusions.

### 2.6. Risk-of-Bias and Study Quality Assessment

Two reviewers independently assessed the risk of bias and methodological quality of clinical studies reporting patient outcomes. Randomized controlled trials were evaluated using the Cochrane Risk of Bias 2 (RoB 2) tool, and non-randomized clinical studies were assessed using the corresponding Joanna Briggs Institute (JBI) critical appraisal checklists according to study design. Dosimetric, in silico, phantom, technical, and protocol studies were not subjected to formal risk-of-bias assessment because conventional clinical risk-of-bias tools were not considered applicable to these study designs. Any disagreements were resolved through discussion and consensus between the two reviewers.

### 2.7. Summary of Searched Manuscripts by Systematic Review

PubMed was used to identify 904 candidate papers. After screening the titles and abstracts and excluding 762 articles, 142 papers remained. After a full-text review of the remaining papers, 108 were deemed to meet the inclusion criteria and were selected for this review. An additional 19 eligible studies related to HyperSight were identified through hand searching. Thus, a total of 127 studies were included in this review. This process is illustrated graphically in the PRISMA flowchart (Figure 1).

### 2.8. Risk of Bias and Overall Study Quality

Of the included studies, 12 clinical studies reporting patient outcomes underwent formal risk-of-bias or methodological quality assessment (Supplementary Table S2). These included one randomized phase II trial and 11 non-randomized clinical studies. Overall, the clinical evidence was predominantly derived from small, single-center, non-randomized studies, with limited comparative data and relatively short follow-up periods. Accordingly, evidence supporting improvements in clinical outcomes remains limited. Across the broader evidence base, improvements in target coverage and/or OAR sparing were consistently reported across multiple disease sites; however, much of this evidence was derived from dosimetric, in silico, phantom, or technical studies that were not subjected to formal risk-of-bias assessment. The available clinical studies primarily evaluated toxicity, treatment response, and short-term oncological outcomes, whereas evidence regarding long-term survival, local control, and patient-reported outcomes remained limited (Supplementary Table S2).

## 3. Workflow of CBCT-Guided oART Using ETHOS^TM^ Therapy

### 3.1. Standard Procedural Steps

**Figure 2:**
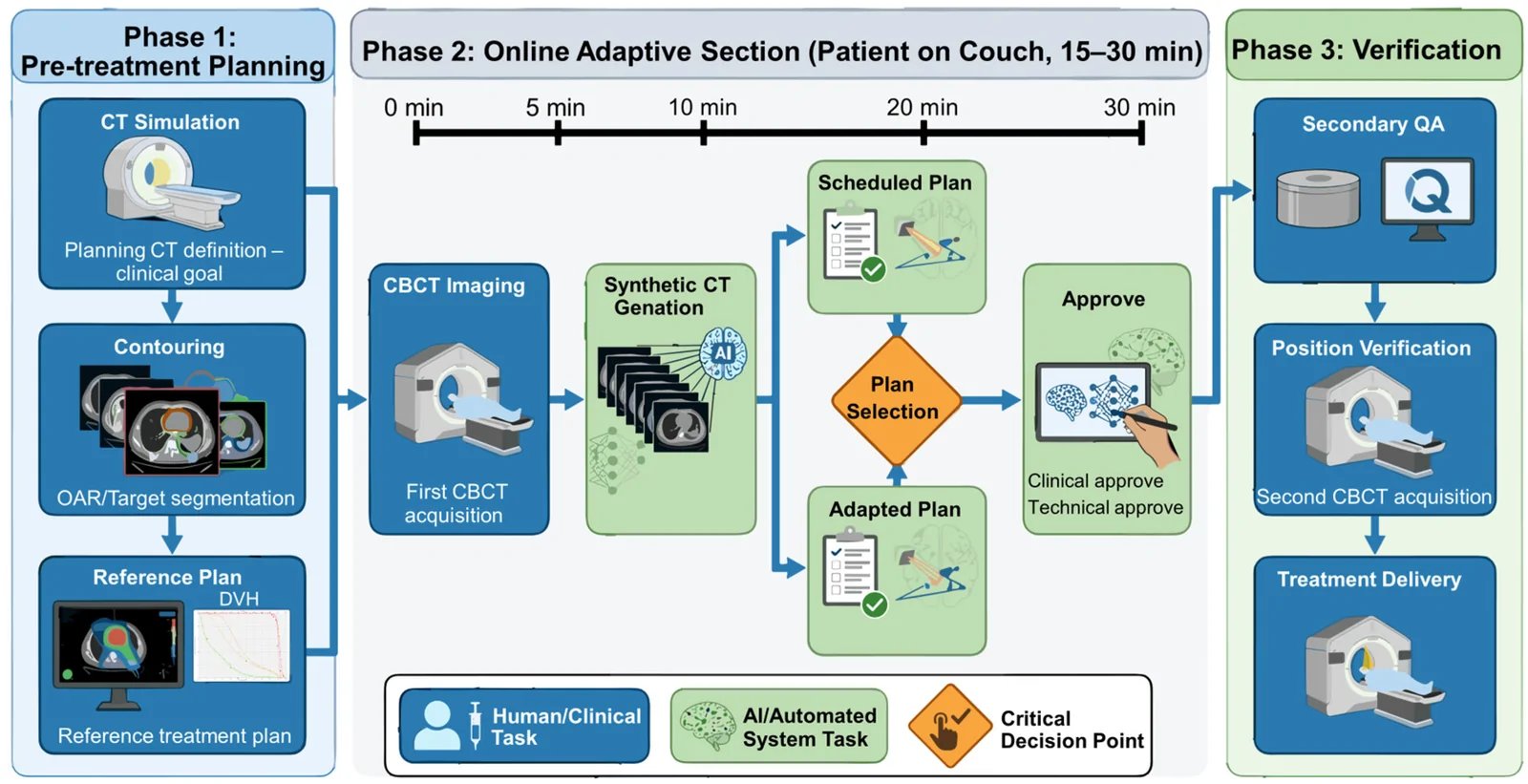
Overview of the clinical workflow for CBCT-guided oART tailored to the ETHOS systems.

The CBCT-guided oART workflow implemented in systems such as the ETHOS platform consists of sequential on-couch processes that combine automated computation and real-time clinical decision making. The workflow can be divided into three phases: pretreatment planning, online adaptive processing, and verification. This section describes the workflow of the ETHOS system (Figure 2).

The online adaptive phase includes the following steps: (I) patient setup, (II) acquisition of the first CBCT, (III) review and editing of OARs (IV) target review and propagation, (V) plan generation, (VI) plan selection, (VII) online quality assurance (QA), (VIII) acquisition of a second verification CBCT, and (IX) treatment delivery [6,7,8]. Steps III–VI are specific to adaptive RT (ART) and are performed while the patient remains on the treatment couch. These steps are performed within a limited time window, which is typically 15–30 min.

### 3.2. Artificial Intelligence (AI)-Driven OARs and Target Segmentation

A key component of the ETHOS oART workflow is the use of AI for rapid contour generation. After CBCT acquisition, AI models generate contours for OARs, particularly for influencer structures such as the bladder and rectum. These structures exhibit substantial day-to-day anatomical variations and have a strong effect on target positioning and dose distribution.

In the ETHOS workflow, OARs are generated and reviewed during the influencer review step. These influencer structures are edited by radiation therapists (RTTs) and/or radiation oncologists and are used to guide subsequent adaptation processes. In the target review step, the system allows either deformable image registration (DIR) or the direct propagation of structures from the planning CT to daily CBCT. When DIR was applied, a deformation vector field was generated to map the planning CT anatomy to the CBCT of the day. This enables the deformation and transfer of target volumes, including gross tumor volume (GTV) and clinical target volume (CTV), onto the daily anatomy. Alternatively, structure propagation can be selected to preserve the original planning CT geometry without deformation, depending on anatomical stability and clinical preference.

In most reported clinical scenarios, AI-generated contours are clinically acceptable with minor manual adjustments for pelvic and thoracic sites, although performance remains dependent on CBCT image quality and the anatomical region [9,10,11].

### 3.3. Plan Generation

Following contour verification, the ETHOS system generates two candidate plans on the synthetic CT derived from DIR: a scheduled plan and an adapted plan [12,13,14].

The scheduled plan represents the original reference plan that was recalculated on the anatomy of the day without re-optimization. By contrast, the adapted plan is a newly optimized plan generated using predefined clinical goals and priority-based constraints within the ETHOS intelligent optimization engine [12,13]. In most implementations, the beam geometry is preserved from the reference plan, the fluence is re-optimized, and the dose is recalculated using a grid-based Boltzmann solver.

Both plans were evaluated using dose–volume metrics and a predefined scorecard that summarized the target coverage and OAR constraints [7]. The adapted plan is selected in the majority of fractions (approximately 90–95%) primarily because of improved target coverage and reduced OAR dose [7,14].

Across multiple cancer sites, the adapted plans consistently demonstrated improved dosimetric performance compared with scheduled plans. These include higher target coverage metrics (e.g., PTV D_95%_–D_99%_) and reduced high-dose regions while maintaining or modestly improving OAR sparing [13,15]. For example, in cervical cancer, adapted plans improved the PTV D_98%_ and reduced bowel and bladder doses per fraction, thus leading to selection in 95% of treatments [14].

The plan selection step is a critical decision point in the workflow. It requires real-time evaluation of the trade-offs between target coverage and OAR sparing and is typically performed by a clinical team with physicist-led assessment and physician oversight depending on institutional practice [7].

### 3.4. QA and Intrafractional Monitoring

Considering that the adapted plan was generated in real-time, independent verification was required before delivery. Online QA is commonly performed using secondary dose calculation software (e.g., Mobius3D), with a typical acceptance criterion of gamma passing rate ≥ 95% at 3%/2 mm [7].

To account for intrafractional anatomical changes during the adaptive process (typically 15–30 min), a second CBCT scan was performed immediately before the treatment. This verified that the anatomy remained consistent with that of the selected plan. If a significant deviation is observed, plan reassessment is required.

Additional motion monitoring methods, such as optical surface imaging, may be used to continuously track patient positions without additional radiation exposure; however, these steps are not intrinsic to all ETHOS workflows [7,8].

### 3.5. Summary

The CBCT-guided oART workflow using the ETHOS system integrates AI-driven contouring, DIR, automated plan generation, and online verification into a clinically feasible on-couch adaptive process. Although recent advances in automation have improved the practicality of online adaptation, the workflow still requires manual review and multidisciplinary decision making to ensure contour accuracy, plan appropriateness, and anatomical consistency before treatment delivery.

## 4. Effectiveness of the ETHOS HyperSight^TM^ CBCT Imaging System

HyperSight is an enabling imaging platform for ETHOS online adaptive radiotherapy (oART), rather than a simple hardware upgrade. Daily cone-beam computed tomography (CBCT) supports anatomy-of-the-day assessment, target and organ-at-risk review, and adaptive plan selection. Its image quality therefore affects both geometric verification and the reliability of the adaptive process. Current evidence indicates that HyperSight improves several limitations of conventional on-board CBCT, including image noise, artifact burden, Hounsfield unit (HU) fidelity, acquisition speed, and anatomical coverage [4,16,17,18,19,20].

Patient and phantom studies consistently demonstrate improved image quality. Robar et al. showed that 6 s HyperSight CBCT acquired during breath-hold produced fewer artifacts than conventional TrueBeam CBCT while maintaining HU values close to those of simulation CT across major tissue classes [4]. Kim et al. reported higher contrast-to-noise ratios, lower image noise, and CT-number calibration errors that approached those of a full CT scanner in CBCT-for-planning mode [16]. Haertter et al. further showed that many HyperSight protocols met most American College of Radiology CT accreditation benchmarks, supporting image quality that approached diagnostic CT standards in benchmark testing and exceeded that of earlier linac-based CBCT systems [17]. A broader technical evaluation also confirmed favorable contrast, HU constancy, and noise characteristics [19]. These gains are clinically relevant because they improve the visibility of soft tissue and anatomical boundaries during contour review.

However, image quality is not uniform across all acquisition conditions. Zhao et al. showed that performance depends on exposure, patient-equivalent size, and field of view, indicating that protocol selection remains important despite improved detector and reconstruction technology [18]. Lustermans et al. similarly reported that HyperSight improved extended field-of-view performance and CT-number robustness across phantom sizes, although quantitative accuracy outside the standard field of view remained inferior to that of fan-beam CT [20]. HyperSight therefore reduces, but does not eliminate, the need for protocol optimization and site-specific quality assessment.

These advantages are particularly relevant in patients with metal implants. Phantom studies demonstrated that the HyperSight metal artifact reduction (MAR) algorithm reduced artifact magnitude and volume around high-density materials and hip prostheses compared with iterative CBCT Acuros reconstruction [21]. Prospective clinical data in head and neck cancer also showed reduced oral cavity artifacts and improved soft-tissue HU accuracy and contrast relative to conventional TrueBeam CBCT [22]. However, MAR may reduce image uniformity when metal artifacts are not the dominant limitation, and Acuros reconstruction may remain preferable in such settings [22]. Reconstruction should therefore be selected according to the primary imaging problem rather than applied uniformly.

HyperSight also offers a practical workflow advantage. Online adaptation is time sensitive, and image quality is clinically useful only when it can be obtained within a feasible treatment session. HyperSight acquires CBCT images in approximately 6 s and provides broader anatomical coverage than earlier ring-gantry systems [4,16]. This rapid acquisition facilitates repeated imaging within the adaptive workflow. In prostate cancer, Kunnen et al. reported improved visualization of the prostate, bladder, rectum, and seminal vesicles, and 99% of HyperSight scans were considered suitable for online adaptation [23]. Schmidt et al. found that initial HyperSight CBCT provided tissue visualization comparable to planning CT during adaptive prostate stereotactic body radiotherapy [24]. Nevertheless, contour sharpness and delineation confidence decreased, and assessment time increased, across fractions. These changes were attributed mainly to treatment-related tissue effects rather than deterioration in technical image quality [24]. Thus, stable image quality does not necessarily ensure constant contouring difficulty throughout treatment.

The value of rapid acquisition is site-dependent. Koo et al. showed that 6 s CBCT accurately localized lung targets during regular and moderately irregular breathing, whereas highly irregular breathing remained challenging [25]. Motion-phantom studies similarly demonstrated that rapid acquisition can introduce target-shape and target-position discrepancies depending on motion amplitude, motion period, and the respiratory phase sampled during the scan [19]. HyperSight improves the practicality of thoracic imaging, but respiratory motion remains an important source of uncertainty.

A further strength of HyperSight is its quantitative performance for adaptive dose assessment. Conventional CBCT has historically been limited by HU inaccuracy and artifacts, which reduce confidence in direct dose calculation. Phantom studies showed that HyperSight-based calculations agreed more closely with CT-based calculations than those performed on earlier Halcyon CBCT and also demonstrated favorable delivery verification results [26,27]. Patient and near-patient studies extended these findings across thoracic, upper abdominal, prostate, pelvic, and breast treatment plans [28,29,30]. Accuracy was generally more consistent in anatomically stable sites, whereas lung cases remained more sensitive to respiratory motion and low-density artifacts [29].

The reconstruction method is a major determinant of quantitative accuracy. Dusi et al. showed that Acuros-reconstructed HyperSight CBCT achieved HU agreement within 28 HU of planning CT across all phantom inserts, whereas Feldkamp–Davis–Kress deviations reached 284 HU [31]. Acuros also produced significantly better gamma passing rates in pelvic cases and maintained stable HU values across 20 prostate CBCT sessions, with standard deviations of 5.0 HU for bone and 4.2 HU for bladder [31]. These findings support the reproducibility of HyperSight-based quantitative imaging over the treatment course. However, the current evidence does not establish HyperSight as a universal replacement for planning CT. Rather, it supports greater confidence in adaptive dose assessment when appropriate acquisition protocols and reconstruction methods are used.

Beyond dose calculation, preliminary evidence suggests that HyperSight may support longitudinal quantitative imaging. Cvachovec et al. reported high stability of HyperSight CBCT-derived radiomic features during adaptive prostate stereotactic body radiotherapy, particularly in organs at risk, where 93.0% of features had an intraclass correlation coefficient greater than 0.9 [32]. This application remains exploratory and is not part of routine ETHOS workflows, but it indicates that daily CBCT may provide reproducible quantitative information in addition to anatomical guidance.

Overall, the evidence is strongest for improved technical image quality, rapid acquisition, and more reliable quantitative dose assessment. In contrast, direct effects on workflow burden, eligibility for adaptation, and clinical outcomes remain less established. Most studies are phantom-based or involve small, single-institution cohorts, and performance varies with anatomical site, motion, acquisition protocol, and reconstruction method. HyperSight should therefore be regarded as a stronger imaging foundation for ETHOS oART rather than a technology that eliminates all imaging-related uncertainty. Prospective multicenter studies are needed to determine whether these technical advantages translate into more efficient adaptation and improved patient outcomes.

## 5. CBCT-Guided oART for Thoracic Tumors

The dosimetric advantages of CBCT-guided oART for thoracic tumors have been demonstrated in several studies, and early signals of clinical utility have begun to emerge. The representative evidence for esophageal, breast, and lung cancers is summarized below.

### 5.1. Esophageal Cancer

In an early dosimetric analysis using an emulator, the magnitude of the benefit varied among patients, with a subset showing a statistically significant reduction in lung and heart doses [33]. Regarding the clinical implementation, Bachmann et al. reported their initial experiences with CBCT-guided oART [34]. CTV modification was required in 59% of fractions, and the adapted plan was selected in 99% of treatment sessions, with a median actual session time of 28 min. Compared with the scheduled plan, the adapted plan reduced the mean heart dose by 9.5% and lung V_20Gy_ by 16.9% while also improving the target coverage. The same group later reported the clinical outcomes of neoadjuvant concurrent chemoradiotherapy using oART, including grade ≥3 toxicities and pathological major response (pMR) [35]. In this study, the adapted plan reproducibly and significantly reduced the mean heart dose and lung V_20Gy_. Acute grade ≥3 toxicities occurred in 27% of patients, and pMR was achieved in 69.2%; however, no significant association was demonstrated between target dose parameters and either toxicity or pMR. Therefore, in esophageal cancer, oART consistently improves heart and lung sparing while maintaining target coverage, although its effect on clinical outcomes remains uncertain.

### 5.2. Breast Cancer

An emulator-based study on breast cancer showed that the adapted plan improved the PTV coverage while maintaining comparable lung, heart, and contralateral breast doses [36]. A retrospective study evaluating the adequacy of CBCT-guided oART for accelerated partial-breast irradiation (APBI) further supported its use [37]. This study showed that the lumpectomy cavity decreased by a median of 16.0% between the simulation and first fraction and that the adapted plan was selected in 86% of fractions, thus indicating that oART can account for rapid postoperative cavity changes. In another study investigating the clinical feasibility of APBI, the adapted plan was used in 90% of the fractions [38]; only three patients developed grade 2 adverse events, and no recurrences were observed. These findings suggest that oART in APBI may offer benefits beyond dosimetric improvement, including minimal toxicity and favorable early oncological outcomes.

Given that breast cancer is a common indication for RT, workflow optimization is an important factor for broader implementation. A prospective study evaluating an RTT-led workflow for APBI showed that contours and plans modified by RTTs under the supervision of a medical physicist were highly concordant with physician-revised versions, with a mean Dice coefficient of 0.96 for the tumor bed and only minimal differences in PTV coverage [39]. Physician involvement time was reduced by 12 min per fraction without compromising treatment quality, thus supporting task redistribution to improve clinical implementation, particularly in APBI.

In whole-breast irradiation, although the effect of postoperative cavity changes is smaller, oART may still help address setup variations, daily changes in breast contour, and uncertainties in arm positioning. In a prospective single-arm study, the on-couch time was 13 min, and no grade ≥3 toxicities were observed [40]. However, contour propagation remained insufficiently accurate for nodal targets such as the internal mammary chain and clavicular lymph nodes, thus indicating that a final physician review is still required [37].

Overall, the clinical feasibility of oART for APBI was supported. However, in more complex cases, further investigations are required to determine the extent to which automation can be reliably implemented.

### 5.3. Lung Cancer

CBCT-guided oART appears particularly promising for SBRT in centrally or ultracentrally located lung tumors and for intensity-modulated RT (IMRT) in locally advanced disease.

In SBRT for centrally located lung tumors, the risk of severe toxicity is higher than that for peripherally located lung tumors because of their proximity to the mediastinal OARs. Accordingly, oART has attracted attention as a strategy for reducing OAR doses while maintaining adequate target coverage. In an emulator-based simulation study of SBRT for ultracentrally located thoracic diseases, CBCT-guided oART was feasible in 97.1% of fractions [41]. When the scheduled plan was recalculated on the anatomy of the day, dose-constraint violations were observed in 62.9% of fractions; by contrast, the adapted plan met all dose constraints in 97.1% of fractions, with an estimated mean workflow time of 28.2 min. To evaluate the clinical outcomes, a phase II single-arm, single-institutional trial of deep-inspiration breath-hold combined with daily oART for centrally located lung tumors is currently ongoing [42]. This approach is expected to increase lung volume and the distance between the tumor and mediastinal OARs. These results clarify the clinical value of oART in central lung tumors.

In locally advanced lung cancer, anatomical changes during the treatment period, such as tumor shrinkage and atelectatic changes, can substantially affect the dose distribution. Studies have shown that scheduled and adapted plans improve target coverage and reduce excessive doses compared with the original reference plan [11]. According to these findings, daily oART may enable both isoeffective treatment and isotoxic dose escalation. Compared with non-adaptive IGRT, isoeffective oART maintained PTV coverage while reducing lung V_20Gy_ and heart V_5Gy_; under isotoxic conditions, a median 10-Gy GTV dose escalation was achievable without increasing lung or heart dose [43]. Therefore, in locally advanced lung cancer, oART could facilitate treatment intensification and optimization, with the potential to improve oncological outcomes without increasing toxicity.

In locally advanced lung cancer, where RT is typically delivered over 6 weeks, the optimal frequency of plan adaptation remains a key practical issue. Although daily adaptation provides the most consistent improvements in target coverage and OAR sparing [44], its routine use throughout the entire treatment course requires substantial time and human resources. A simulation study suggested that weekly adaptation may offer a more efficient alternative to the traditional replanning process, with greater reductions in heart, esophageal, and lung doses in patients whose tumors have shrunk by >10% [45]. These findings suggest that the magnitude of tumor regression may help guide the timing of oART.

### 5.4. Summary

Across esophageal, breast, and lung cancers, CBCT-guided oART has consistently demonstrated dosimetric benefits, and the early signals of clinical utility are beginning to emerge. However, current evidence is limited by small sample sizes, retrospective and/or single-institution study designs, short follow-up periods, immature clinical outcome data, and substantial demands on time and human resources. Given that these thoracic malignancies are common indications for RT, prospective multicenter studies are warranted to better define the clinical role of CBCT-guided oART, including optimal adaptation timing, appropriate patient selection, automation reliability, and resource allocation efficiency.

## 6. CBCT-Guided oART for Upper Abdominal Malignancies

CBCT-guided oART has been investigated for several upper abdominal malignancies, where daily anatomical variations, respiratory motion, and proximity to radiosensitive organs can complicate treatment delivery. The available evidence is summarized below by disease site: liver tumors, pancreatic cancer, gastric mucosa-associated lymphoid tissue (MALT) lymphoma, and abdominal oligometastases.

### 6.1. Liver Tumors

In liver tumors, CBCT-guided oART improved target coverage, treatment accuracy, and OAR sparing compared with scheduled plans. Adapted plans achieved PTV coverage goals (PTV V100% ≥ 95%) more frequently than scheduled plans (92% vs. 64%, *p* = 0.042); in fractions with target-OAR overlap, ART achieved adequate target coverage in 87% of fractions compared with only 33% of scheduled plans and reduced the maximum dose to adjacent gastrointestinal organs, including the stomach and duodenum [46].

From a geometric and safety perspective, CBCT-guided oART significantly reduced high-priority OAR constraint violations from 4.2% to 0.9% (*p* < 0.001) and demonstrated accurate target localization, with mean geometric errors of 4.3 ± 2.1 mm; this indicated that this method supported reliable treatment delivery and improved treatment precision [47].

### 6.2. Pancreatic Cancer

In pancreatic cancer, CBCT-guided oART consistently improved the target dose coverage despite substantial interfractional anatomical variations. The adapted plans achieved PTV coverage goals (PTV V95% > 95%) in 97% of fractions [48]. Another clinical study showed that ART improved PTV V98% from 89.7% to 96.3% (*p* < 0.001) and PTV V95% from 89.7% to 96.3% (*p* = 0.031) while reducing duodenum D0.5cc from 33.6 Gy to 28.8 Gy (*p* < 0.001), small bowel D0.5cc from 31.4 Gy to 30.2 Gy (*p* = 0.037), and stomach D0.5cc from 41.2 Gy to 33.5 Gy (*p* < 0.001) [49].

A simulation study using clinical breath-hold CBCT datasets further supported these findings and showed that adaptive planning respectively increased GTV D98% and CTV D98% in 60.1% and 74.0% of the fractions and respectively reduced stomach and duodenum constraint violations from 28.5% and 29.1% with scheduled plans to 0% with adapted plans [50].

Moreover, oART may support dose escalation for pancreatic cancer. ART enabled isotoxic dose escalation while maintaining OAR constraints, thus suggesting a potential improvement in the therapeutic ratio [51].

### 6.3. Gastric MALT Lymphoma

In gastric MALT lymphoma, CBCT-guided oART improved target coverage and reduced normal tissue exposure compared with scheduled plans. ART significantly increased CTV D98% from 94.7% to 98.6% and D95% from 97.3% to 99.2% (*p* < 0.001) while reducing the kidney and spinal cord doses [52]. Additional analyses also showed improvements in CTV D50%, D98% and Dmean, together with lower liver and kidney doses, compared with non-ART [15].

Clinical feasibility and plan robustness have also been evaluated for gastric MALT lymphomas. oART was completed in approximately 45 min per fraction, thus indicating clinical feasibility despite the additional time required for repeated breath-hold image acquisition and treatment delivery. Although CBCT-guided oART systems do not provide continuous intrafraction tumor visualization during irradiation, the verification of CBCT acquired immediately before and after treatment delivery showed no significant degradation in target coverage or OAR dose compared with the adapted plan, thus indicating that this method supported robust dose delivery despite intrafraction motion and breath-hold variability [15,52].

CBCT-guided oART may also allow margin reduction in gastric MALT lymphomas. An isotropic margin analysis demonstrated that PTV margins could be reduced from 25 mm to 14 mm with oART, thus reflecting the improved compensation for interfractional gastric motion [53]. Further geometric analysis incorporating intrafraction deformation showed that anisotropic margin design could enable direction-dependent margin optimization, with required PTV margins ranging from 9.7 mm to 13.1 mm to ensure target coverage in 90% of fractions [54].

### 6.4. Abdominal Oligometastases

In abdominal oligometastases, CBCT-guided oART improved target coverage, OAR constraint compliance, and treatment deliverability. Adaptive RT improved target coverage in 63% of fractions based on GTV V100% and in 50% of fractions based on D95% while reducing OAR constraint violations from 75% of scheduled plans to 5% with oART [55]. In another analysis of abdominal lymph node oligometastases, oART improved PTV Dmean and D98%; reduced OAR dose metrics, including Dmax and D0.5cc; increased the proportion of plans meeting both target and OAR constraints from 34.4% to 97.1%; and enabled treatment delivery in 97.1% of cases [56]. These findings suggest that CBCT-guided oART may expand the feasibility of ablative or dose-escalated treatment strategies in selected cases of abdominal oligometastases.

### 6.5. Summary

Overall, CBCT-guided oART improved target coverage, reduced the OAR dose, improved dose-constraint compliance, supported dose escalation, and enhanced geometric precision across upper abdominal malignancies. However, most available data are dosimetric or feasibility focused, with limited clinical outcome data regarding survival benefits and toxicity reduction. Further prospective studies are needed to confirm the clinical effect of CBCT-guided oART for upper abdominal malignancies.

## 7. CBCT-Guided oART for Pelvic Malignancies

CBCT-guided oART in the pelvic region has been predominantly reported in genitourinary [57,58,59,60,61] and gynecologic malignancies [62,63,64,65,66,67,68]. The primary role of oART in the pelvic region is to accurately account for day-to-day variations (interfractional errors) in the shape and position of targets and normal tissues, such as the bladder and bowel. By adapting the treatment plan to the daily anatomy, oART aims to improve the target dose coverage while reducing doses to the surrounding normal tissues.

### 7.1. Genitourinary Malignancies

CBCT-guided oART has been predominantly used in definitive RT for nonmetastatic prostate cancer. Several dosimetric studies have demonstrated improvements in dose–volume histogram (DVH) parameters with the use of oART, including reductions in dose to normal tissues and enhanced target dose coverage [9,69,70,71,72,73,74,75,76,77]. These dosimetric data suggest the potential to reduce PTV margins without compromising the target coverage [70,74,75,77], thus potentially reducing radiation-induced toxicity. In addition, its advantages have recently been investigated in the setting of postoperative RT to the prostate bed, in which significant improvements in target dose coverage were demonstrated with oART compared with non-adaptive IGRT [73]. Intrafractional prostate motion is a major source of geometric uncertainty that can impair treatment accuracy [78]. Prolonged treatment time is a critical consideration in the clinical implementation of oART for prostate RT. Although the dosimetric benefit of oART over non-adaptive approaches was reportedly maintained despite the discrepancies between planned and delivered doses due to intrafractional errors in clinical practice settings [72], the optimization of workflow to reduce treatment duration remains an important issue in its clinical implementation.

Several studies have reported short-term outcomes, primarily focusing on acute toxicity [57,58,60]. According to the retrospective comparative analysis by Zhu et al., who compared the rate of acute toxicities between daily oART (n = 81) and non-adaptive radiation therapy (n = 77) among patients treated with moderately hypofractionated IMRT (60 Gy in 20 fractions), CBCT-guided oART was significantly associated with reduced acute gastrointestinal toxicity compared with non-ART (odds ratio [OR], 0.45; 95% confidence interval [CI], 0.27–0.73; *p* = 0.001); by contrast, no significant reduction was observed for acute genitourinary toxicity (OR, 0.65; 95% CI, 0.40–1.03; *p* = 0.07) [58]. Muscle-invasive bladder cancer (MIBC) is another major genitourinary malignancy for which the merits of oART are highly expected [59,61,79,80,81,82]. Given the substantial interfractional variability in bladder shape and volume and adjacent OARs, such as the bowel, oART is considered particularly well suited. Considering that MIBC predominantly affects elderly patients [83], RT is often employed not only as part of tri-modality therapy for bladder preservation [84] but also as a treatment option for patients in whom more invasive therapies are not feasible [85,86]. The advantages of oART are particularly relevant in the setting of definitive RT for medically frail individuals, particularly those who have difficulty adhering to treatment instructions, such as bladder filling or voiding protocols. According to a dosimetric analysis of CBCT-guided oART in two elderly patients with MIBC and dementia who were deemed poor candidates for surgery or systemic therapies, oART significantly improved the dose distribution compared with scheduled non-adaptive plans [87]. Specifically, a significant reduction in high-dose bowel exposure (V_90%_) was achieved in both patients without impairing target dose coverage; the median bowel V_90%_ with oART and non-adaptive plans in the first patient were 1.25 and 24.78 cc (*p* < 0.001), respectively, and those in the second patient were 0.13 and 9.6 cc (*p* < 0.001), respectively. Both patients completed the treatment course with manageable acute toxicities, and no grade ≥ 2 events were observed. This study offers promising results regarding the utility and feasibility of oART for MIBC even in elderly or medically fragile populations.

### 7.2. Gynecologic Malignancies

Clinical data on CBCT-guided oART in this field have been predominantly reported in definitive RT for cervical cancer [62,63,64,65,66,68]. Given that the uterus exhibits substantial interfractional variability, oART is expected to offer considerable advantages, including improvements in DVH parameters and the potential for PTV margin reduction [10,14,88,89,90,91,92]. According to the dosimetric evaluation, which compared daily oART with conventional IGRT in definitive RT for cervical cancer, daily oART enabled the substantial reduction in PTV margins for the cervix/uterus/GTV (0.5 cm in oART vs. 1.5 cm in conventional IGRT) and those for the parametria/vagina (0.5 cm in oART vs. 1.0 cm in conventional IGRT) with significant improvements in CTV coverage and OAR sparing [88]. Similarly, according to a prospective study of daily oART for cervical cancer, in which all patients received daily CBCT-guided oART (prescribed at 50.4 Gy in 28 fractions) with concurrent weekly chemotherapy followed by brachytherapy, the adapted plans showed superior target coverage and improved OAR doses to the scheduled plan [63]. Overall, the incidence of acute toxicity following oART was relatively low. Specifically, grades 1, 2 and 3 acute gastrointestinal toxicities occurred in 26%, 19% and 4% of patients, respectively, among which diarrhea was the most common gastrointestinal toxicity. Only grade 1 acute genitourinary toxicity was observed in 7% of patients. No grade ≥ 4 acute toxicities were observed. Currently, a phase III randomized controlled study, namely, SWIFT-1, is ongoing to evaluate the non-inferiority of moderately hypofractionated RT to conventional fractionated RT, in which oART is used in the hypofractionated RT arm [93]. The potential benefits of oART in this field will be further evaluated in this clinical trial, and the results are anticipated.

### 7.3. Rectal Cancer, Anal Cancer, and Other Pelvic Malignancies

Regarding other pelvic malignancies, oART has also been investigated in gastrointestinal cancers, specifically rectal and anal cancers [94,95,96,97]. As observed in other pelvic cancers, oART has the potential to improve dose distribution and reduce the dose to the surrounding normal tissues; this would be beneficial for reducing RT-induced toxicities because RT for these tumors is performed in combination with chemotherapy. According to the dosimetric analysis of oART for locally advanced rectal cancer, the use of CBCT-guided oART significantly reduced the rectal boost PTV margines [94]. Specifically, the rectal boost PTV margins for non-adaptive vs. oART were 14.2 mm vs. 3.3 mm in the antero-posterior axis, 5.0 vs. 3.2 mm in the left–right axis, and 12.3 vs. 8.7 mm in the supero-inferior axis, respectively. However, considering that RT for these tumors is commonly delivered with conventional fractionation over a longer treatment course, it is important to maintain a balance between dosimetric benefit and practical feasibility.

### 7.4. Summary

Among genitourinary, gynecologic, and gastrointestinal malignancies in the pelvic region, CBCT-guided oART has demonstrated dosimetric advantages, particularly in accounting for substantial interfractional anatomical variations. Although the current evidence remains limited by small sample sizes and relatively short follow-up periods, early clinical experience suggests the potential feasibility and utility of CBCT-guided oART in this field, including reduction in acute toxicity in selected settings and improved treatment precision for anatomically mobile targets. Therefore, further studies are warranted to better define the clinical role of CBCT-guided oART, including optimal patient selection, workflow efficiency, and balance between dosimetric benefits and practical feasibility.

## 8. CBCT-Guided oART for Head and Neck Cancers

In the head and neck cancer setting, eight studies were included, comprising one randomized phase II trial comparing oART with conventional IGRT [98], six studies that primarily performed dosimetric comparisons between adapted and scheduled plans [13,99,100,101,102,103], and one retrospective methodological study focusing on the development and validation of a dosimetrically triggered oART decision-support protocol [104]. The sample sizes ranged from approximately 10 to 69 patients, with most cohorts consisting of patients undergoing definitive RT, often in combination with concurrent chemotherapy. The prescribed doses were typically 64–70 Gy delivered in 32–35 fractions. The frequency of oART varied widely, ranging from daily adaptation to more selective approaches with only 1–3 adaptations during treatment. Another study further explored the application of oART in QUAD-shot RT [99].

Dosimetric analyses have consistently demonstrated the advantages of oART. All six studies comparing adapted and scheduled plans reported improvements in target-related dose metrics and indicated enhanced target coverage with adaptation [99,100,101,102,103]. These findings support the fundamental concept of oART, namely, the ability to re-optimize the dose distribution in response to anatomical changes during treatment, such as tumor shrinkage and weight loss. This rationale is particularly relevant for head and neck cancers, where such changes are often substantial. Likewise, improvements in OARs were observed. However, dose evaluation methods varied across studies, with some reporting cumulative treatment-course doses and others reporting per-session values. For consistency, per-session estimates were converted to the corresponding dose for a 35-fraction treatment course. The mean doses to the ipsilateral and contralateral parotid glands were evaluated in three studies, with changes ranging from −8.3 to +0.06 Gy and from −8.1 to +0.99 Gy, respectively [13,101,103]. The maximum spinal cord dose was evaluated in two studies and demonstrated reductions ranging from −3.36 to −1.16 Gy [100,103]. Overall, OAR doses were generally maintained or reduced; however, the magnitude of benefit varied across studies, likely reflecting differences in patient anatomy and extent of tumor shrinkage.

Guberina et al. reported the relationship between anatomical changes and margin design in dosimetric analysis [102]. By using equivalent uniform dose-based cumulative dose evaluation, the minimum PTV margin required to maintain adequate target coverage was 5 mm for the scheduled plans and could be reduced to 3 mm for the adaptive plans. These findings suggest that conventional margin strategies may be overly conservative and that oART may enable margin reduction by compensating for interfractional anatomical variations.

Furthermore, in a randomized phase II trial, oART demonstrated superior OAR sparing to IGRT, thus suggesting the potential of oART to better maintain planned dose distributions in clinical practice [98]. Despite these consistent dosimetric improvements, evidence supporting their corresponding clinical benefits remains limited. In a randomized trial, oART was non-inferior to IGRT in terms of 2-year overall survival, progression-free survival, and recurrence outcomes, thus indicating that adaptation does not compromise oncologic efficacy. Regarding toxicity, no significant difference was observed in the primary endpoint of xerostomia as measured by the XQ score. However, the incidence of acute dermatitis was significantly reduced, and a trend toward reduced mucositis was observed, although this did not reach statistical significance [98]. In a prospective study by Blumenfeld et al., the incidence of grade 3 dermatitis and mucositis was reported to be 4.5%, with no grade 4 and 5 adverse events observed, thus suggesting a favorable toxicity profile compared with previous reports [100]. However, the current body of evidence has largely focused on dosimetric endpoints, and a direct relationship between OAR dose reduction and clinically meaningful toxicity reduction has not yet been established. Further prospective clinical studies are required to address this issue.

From a practical standpoint, workflow considerations remain key factors in the implementation of oART. In head and neck cancers, the in-room time per fraction ranges from approximately 34.5 to 45.8 min [13,98,102,103], with 17 to 30 min required for the adaptive process itself [13,98,99,100,103]. Although methodological studies have explored decision-support frameworks, such as dosimetrically triggered protocols, to guide patient selection for oART, prolonged treatment times and increased demands on personnel and infrastructure may represent barriers to broader implementation. In addition, most available studies are based on dosimetric comparisons between adapted and scheduled plans. Although such analyses are valuable for demonstrating theoretical advantages, they may not fully account for real-world factors, such as contouring variability and the effect of prolonged treatment time, including increased intrafractional motion.

Additionally, oART may provide value beyond conventional clinical endpoints by reducing patient burden and environmental effects. In the context of QUAD-shot RT, Liu et al. reported that the implementation of oART enabled the omission of repeat simulation CT scans, thus resulting in reduced patient travel distance and associated CO_2_ emissions [99]. These findings suggest that the benefits of oART may extend beyond dosimetric optimization and encompass patient convenience and sustainability.

CBCT-guided oART provides consistent dosimetric advantages in head and neck cancers, including improved target coverage and reduced doses to the OARs. These characteristics provide a strong theoretical rationale for its use at disease sites that are characterized by substantial anatomical changes during treatment. However, whether these dosimetric benefits translate into clear clinical advantages remains to be fully established. Further prospective clinical data are required to define the true value of oART in this setting.

## 9. Development of CT-Sim-Free oART Workflows

In conventional RT, the CT-sim workflow is a fundamental component. This process includes contouring the target volume and OARs, dose optimization, and physical QA. Depending on the complexity, the transition from simulation to treatment can require 1–2 days for palliative cases and several days to weeks for high-precision RT. These delays can result in disadvantages such as prolonged physical distress, deterioration of quality of life, and extended hospital stays, particularly for patients presenting with pain or neurological symptoms [105]. To overcome these physical and temporal constraints, the feasibility and utility of CT-sim-free oART technology have been increasingly investigated. This review evaluated the current situation and clinical utility of CT-sim-free oART.

Among the 108 oART-related articles identified in this review, 8 (7.4%) evaluated the feasibility or clinical utility of CT-sim-free oART workflows [106,107,108,109,110,111,112,113]. The clinical targets investigated in these studies were bone metastases, rectal cancer, and abdominal malignancies. Nelissen et al. conducted an in silico analysis of 15 patients with bone metastases by using diagnostic CT (dCT) scans and CBCT scans acquired at the time of irradiation [106]. The study simulated a workflow that included the registration of dCT-based pre-plans to CBCT, generation of synthetic CT (sCT), and generation of oART plans. All oART plans met the clinical goals, and dose calculations using CBCT and sCT demonstrated high consistency. These dosimetric data indicated that online adaptive technology enables the on-couch generation and delivery of clinically acceptable treatment plans without prior CT-sim. Subsequently, the FAST-METS study, a prospective observational trial, evaluated the clinical feasibility of a CT-sim-free oART workflow in 43 patients with 47 metastatic bone metastasis lesions [107]. The primary endpoints were dosimetric data and treatment time, with patient satisfaction as the secondary endpoint. All patients received 8 Gy of radiation in a single fraction. The oART plans were implemented in all treatments, significantly improving the target dose coverage compared with the scheduled plans (*p* < 0.005). The median on-couch time was 30 min. Patient satisfaction was high, with 80% of participants expressing a preference for CT-sim-free oART for future treatment. Kejda et al. prospectively assessed the feasibility of CT-sim-free oART utilizing sCT generated from CBCT in 20 palliative cases (23 lesions), including bone metastases and thoracic or abdominal soft-tissue lesions [108]. A simultaneous integrated boost was planned for 18 of the 23 lesions. Despite the complexity of simultaneous integrated boost planning, improvements in target dose coverage were observed, and the median time for the immediate adaptive process was 13.2 min, consistent with previous reports. These findings demonstrate that CT-sim-free oART is a clinically feasible strategy and viable option for patients requiring minimally invasive palliative care. However, these studies highlighted challenges, such as artifacts from daily fluctuations in bowel gas and the resulting decrease in the accuracy of DIR. Particularly in lesions of the lumbar spine and pelvis, such artifacts led to the generation of inaccurate sCT, thus compromising dose calculation reliability and occasionally resulting in the abandonment of the oART workflow. Owing to these uncertainties, prior research has primarily focused on palliative settings and targets, such as the bone, and application to thoracic and abdominal organs remains difficult.

Advances in CBCT imaging quality, particularly high HU fidelity comparable to that of fan-beam CT, have addressed these limitations. Schuurhuizen et al. evaluated the feasibility of CT-sim-free oART by using high-quality CBCT data from 15 patients with stage II–III rectal cancer who received preoperative (chemo) RT [109]. In the simulation delivering the dCT-based plans without adaptation (n = 15), none of the plans met the required dose constraints. Conversely, all adapted plans generated through the online adaptive workflow using high-quality CBCT (n = 5) successfully met the constraints for both target volume coverage and OARs. This research group is currently conducting a prospective clinical trial (MEC 2023-0445) to evaluate the clinical utility of this CT-sim-free oART workflow in patients with rectal cancer undergoing preoperative (chemo) RT, with further anticipated results. Price et al. simulated a CT-sim-free oART workflow (SBRT: 50 Gy in 5 fractions) by using high-quality CBCT with data from five patients with pancreatic cancer and three with abdominal oligometastases [110]. In this study, only the GTV was pre-contoured on the dCT, whereas the other structures were automatically delineated during the oART process. For plan validation, oART plans were compared with initial adaptive plans that utilized the full contouring of the OARs and planning structures on simulation CTs. The median absolute difference in target volume (V_95%_) between the oART plans and the simulation CT-based plans was 2.0%, and the differences in OAR doses ranged from −5.3 Gy to 1.2 Gy. The mean total workflow time was 63 min. These findings indicate that CT-sim-free oART utilizing high-quality CBCT demonstrates technical feasibility even for high-precision RT.

Facilitated by advancements in high-quality CBCT, the feasibility of direct planning workflows on the treatment couch is currently under investigation. MacDonald et al. evaluated this process in silico by creating pre-plans using high-quality CBCT followed by oART [111]. The results demonstrated that the difference between the calculated and measured doses was within 1.6%, successfully passing the external dose audit protocol. By building on these findings, the same group simulated 8-Gy single-fraction oART sessions by using data from 10 patients with spinal metastases [112]. The simulation compared three reference plan models: a standard reference plan based on conventional simulation CT, a generalized template plan (library plan) created using fan-beam CT from a different patient, and a complete dummy reference plan based on a phantom. The oART plans generated from the library and phantom-based reference plans showed no significant differences compared with the standard workflow across all clinical constraints regarding target coverage and OAR dose reduction (*p* > 0.2). The authors concluded that an oART workflow that eliminates the need for patient-specific pre-planning is feasible for palliative RT for spinal metastases. Furthermore, the evaluation of pre-plan-free workflows is expanding to SBRT. Wang et al. assessed the feasibility of a pre-plan-free oART workflow that uses library plans for spinal SBRT [113]. This workflow was simulated using data from five thoracic and five lumbar spine cases with previously acquired high-quality CBCT images. The results demonstrated that all oART plans achieved sufficient target dose coverage while satisfying the dose constraints for the OARs. The quality of the oART plans generated from the library plans was comparable with that of the original reference plans. These findings indicate that direct and immediate dose calculations on images acquired at the treatment console are technically feasible, thus suggesting that CT-sim and patient-specific pre-planning could potentially be omitted.

However, several challenges remain in the clinical implementation of CT-sim-free oART. First, the long-term clinical outcomes have not yet been established. The current findings on CT-sim-free oART are limited to technical feasibility validations and initial dosimetric evaluations in small cohorts. To establish this approach as a standard of care, it is essential to demonstrate its long-term efficacy and safety. Second, the prolonged workflow times pose a hindrance. The entire oART process requires time, thus leading to on-couch times that exceed standard treatment slots. Prolonged immobilization can cause distress, particularly in symptomatic patients. Furthermore, implementing this technology in routine practice requires dedicated staff to remain on the console for real-time plan modification and approval. These onsite requirements reduce machine throughput and increase staff workload. Addressing these challenges requires further enhancing the accuracy of AI-driven auto-contouring and DIR and establishing task-shifting models, such as radiotherapist-led adaptation [114].

In summary, CT-simulation-free oART is a technique that enables the reduction in waiting times for treatment initiation and adaptation to daily anatomical variations. Although technical feasibility has been reported in both palliative and high-precision RT, current evidence remains in an experimental phase, limited to in silico simulations and initial small-scale feasibility studies. Challenges such as the establishment of long-term clinical outcomes and the improvement of workflow efficiency persist for clinical implementation, requiring further studies to evaluate the clinical utility of this approach.

## 10. Conclusions

This review summarizes the dosimetric and clinical benefits of oART for several types of cancer. Furthermore, we showed the general workflow of oART, as well as high-speed and high-quality CBCT, HyperSight and CT-sim-free methods that are expected to be used in the future. We hope that this manuscript will be helpful for introducing oART in several institutions. oART technology is certainly attractive and is expected to improve clinical outcomes in the future; however, various challenges remain regarding its widespread adoption. Further improvements in workflow, reduction in treatment time, accurate contouring by high-precision AI, and high-resolution imaging are necessary. It is also important to increase the number of healthcare professionals who can perform oART, and the development of educational systems for physicians, physicists, and technicians is essential. ETHOS 2.0 is expected to be an attractive update that addresses some of the limitations in the treatment planning of the previous ETHOS version and will enable improved dose concentration in SBRT and direct calculation using high-resolution images [115,116]. While the papers collected for this review focused primarily on ETHOS-based CBCT-guided oART, it is expected to be implemented using various machines and will become more widespread in the future. From 2025 another device (Elekta, Evo^TM^) is also available on the market for oART using CBCT and from 2026 it is also possible to perform oART on other Varian machines (Truebeam^TM^ in connection with RaySearch Laboratories Software).

This review used a systematic review approach to extract comprehensive evidence; however, the study has several limitations. The risks of publication bias, selective reporting, and the predominance of early-adopter institutions utilizing the ETHOS platform cannot be ruled out, because empirical feasibility studies are more likely to be published than failed implementations, potentially leading to an overestimation of the technology’s benefits. In addition, the literature search was limited to PubMed, and potentially relevant studies indexed only in other databases may have been missed. It is warranted that further studies reporting on the strengths and weaknesses of CBCT-guided adaptive radiotherapy will enable a true evaluation of this technology.

Most available evidence of CBCT-guided oART remains dosimetric or feasibility-based rather than demonstrating improvements in overall survival, local control, or patient-reported outcomes. Several retrospective and in silico studies have been conducted, but further prospective studies are warranted to demonstrate the clinical benefit of oART. In the future, the development of oART is expected to lead to the introduction of personalized medicine and improve the clinical outcomes of patients with various cancers.

## Data Availability

The data supporting the findings of this systematic review are available from the corresponding author upon reasonable request.

## Supplementary Materials

The following supporting information can be downloaded at: https://www.mdpi.com/article/10.3390/cancers18172755/s1, Table S1: PRISMA 2020 Checklist; Table S2: Risk-of-bias and methodological quality assessment of clinical studies reporting patient outcomes.

## Abbreviations

The following abbreviations are used in this manuscript:

RT	Radiotherapy
IGRT	Image-Guided Radiotherapy
CBCT	Cone-Beam Computed Tomography
iCBCT	Iterative CBCT
CT	Computed Tomography
FDK	Feldkamp–Davis–Kress
oART	Online Adaptive Radiotherapy
OARs	Organs at Risk
MRI	Magnetic Resonance Imaging
PTV	Planning Target Volume
PRISMA	Preferred Reporting Items for Systematic Reviews and Meta-Analyses
CT-sim	CT Simulation
QA	Quality Assurance
AI	Artificial Intelligence
RTTs	Radiation Therapists
DIR	Deformable Image Registration
GTV	Gross Tumor Volume
CTV	Clinical Target Volume
HU	Hounsfield Unit
MAR	Metal Artifact Reduction
pMR	Pathological Major Response
APBI	Accelerated Partial-Breast Irradiation
SBRT	Stereotactic Body Radiotherapy
IMRT	Intensity-Modulated Radiotherapy
MALT	Mucosa-Associated Lymphoid Tissue
MIBC	Muscle-Invasive Bladder Cancer
DVH	Dose–Volume Histogram
dCT	Diagnostic CT
sCT	Synthetic CT

## References

[1] Bertholet J., Anastasi G., Noble D., Bel A., van Leeuwen R., Roggen T., Duchateau M., Pilskog S., Garibaldi C., Tilly N. (2020). Patterns of practice for adaptive and real-time radiation therapy (POP-ART RT) part II: Offline and online plan adaption for interfractional changes. Radiother. Oncol..

[2] Liu X., Li Z., Yin Y. (2023). Clinical application of MR-Linac in tumor radiotherapy: A systematic review. Radiat. Oncol..

[3] Mao W., Liu C., Gardner S.J., Siddiqui F., Snyder K.C., Kumarasiri A., Zhao B., Kim J., Wen N.W., Movsas B. (2019). Evaluation and clinical application of a commercially available iterative reconstruction algorithm for CBCT-Based IGRT. Technol. Cancer Res. Treat..

[4] Robar J.L., Cherpak A., MacDonald R.L., Yashayaeva A., McAloney D., McMaster N., Zhan K., Cwajna S., Patil N., Dahn H. (2024). Novel technology allowing cone beam computed tomography in 6 seconds: A patient study of comparative image quality. Pract. Radiat. Oncol..

[5] Kalinauskaite G., Künzel L.A., Kluge A., Rubarth K., Dannehl J., Höhne C., Beck M., Zips D., Senger C. (2025). Optimizing workflow for cone beam computed tomography-based online adaptive radiation therapy toward reduced physician involvement. Adv. Radiat. Oncol..

[6] Stanley D.N., Harms J., Pogue J.A., Belliveau J.G., Marcrom S.R., McDonald A.M., Dobelbower M.C., Boggs D.H., Soike M.H., Fiveash J.A. (2023). A roadmap for implementation of kV-CBCT online adaptive radiation therapy and initial first year experiences. J. Appl. Clin. Med. Phys..

[7] Stanley D.N., Covington E., Harms J., Pogue J., Cardenas C.E., Popple R.A. (2023). Evaluation and correlation of patient movement during online adaptive radiotherapy with CBCT and a surface imaging system. J. Appl. Clin. Med. Phys..

[8] Byrne M., Archibald-Heeren B., Hu Y., Teh A., Beserminji R., Cai E., Liu G., Yates A., Rijken J., Collett N. (2022). Varian ethos online adaptive radiotherapy for prostate cancer: Early results of contouring accuracy, treatment plan quality, and treatment time. J. Appl. Clin. Med. Phys..

[9] Branco D., Mayadev J., Moore K., Ray X. (2023). Dosimetric and feasibility evaluation of a CBCT-based daily adaptive radiotherapy protocol for locally advanced cervical cancer. J. Appl. Clin. Med. Phys..

[10] Mao W., Riess J., Kim J., Vance S., Chetty I.J., Movsas B., Kretzler A. (2022). Evaluation of auto-contouring and dose distributions for online adaptive radiation therapy of patients with locally advanced lung cancers. Pract. Radiat. Oncol..

[11] Yoganathan S.A., Khemissi A., Paloor S., Hammoud R., Al-Hammadi N. (2025). An end-to-end quality assurance procedure for ethos online adaptive radiotherapy. J. Med. Phys..

[12] Shi J.J., Meduri K., Liao C.Y., Moon D.H., Avkshtol V., Parsons D., Zhong X., Chen L., Lin M.H., Sher D.J. (2025). Dosimetric advantages of online adaptive radiation therapy for head and neck squamous cell carcinoma: Results from a prospective registry study. Int. J. Radiat. Oncol. Biol. Phys..

[13] Shelley C.E., Bolt M.A., Hollingdale R., Chadwick S.J., Barnard A.P., Rashid M., Reinlo S.C., Fazel N., Thorpe C.R., Stewart A.J. (2023). Implementing cone-beam computed tomography-guided online adaptive radiotherapy in cervical cancer. Clin. Transl. Radiat. Oncol..

[14] Uto M., Iramina H., Iwai T., Yoshimura M., Mizowaki T. (2025). Treatment time and dosimetric advantage in cone beam computed tomography-guided online adaptive radiation therapy considering interfractional and intrafractional changes in patients with gastric mucosa-associated lymphoid tissue lymphoma. Pract. Radiat. Oncol..

[15] Kim E., Park Y.K., Zhao T., Laugeman E., Zhao X.N., Hao Y., Chung Y., Lee H. (2024). Image quality characterization of an ultra-high-speed kilovoltage cone-beam computed tomography imaging system on an O-ring linear accelerator. J. Appl. Clin. Med. Phys..

[16] Haertter A., Salerno M., Koger B., Kennedy C., Alonso-Basanta M., Dong L., Teo B.K., Li T. (2024). ACR benchmark testing of a novel high-speed ring-gantry linac kV-CBCT system. J. Appl. Clin. Med. Phys..

[17] Zhao H., Nelson N., Streitmatter S., Nordin B., Martin T., Price R.G., Kunz J.N., Bhagroo S., Huang Y.J., Nelson G. (2025). Correlation between exposure (mAs) and image quality for a rapid cone-beam CT on a ring gantry linear accelerator. J. Appl. Clin. Med. Phys..

[18] Zhao H., Nelson G., Sarkar V., Oare C., Szegedi M., James S.S., Kunz J., Price R., Huang Y.J. (2025). Comprehensive image quality evaluation and motion phantom studies of an ultra-fast (6-second) cone-beam computed tomography imaging system on a ring gantry linear accelerator. Adv. Radiat. Oncol..

[19] Lustermans D., Fonseca G.P., Taasti V.T., van de Schoot A., Petit S., van Elmpt W., Verhaegen F. (2024). Image quality evaluation of a new high-performance ring-gantry cone-beam computed tomography imager. Phys. Med. Biol..

[20] Yashayaeva A., MacDonald R.L., Robar J., Cherpak A. (2024). Evaluation of a metal artifact reduction algorithm for image reconstruction on a novel CBCT platform. J. Appl. Clin. Med. Phys..

[21] Yashayaeva A., MacDonald R.L., Zhan K., DeGiobbi J., McMaster N., McAloney D., Ward L., Anderson C., LeBlanc M., Best L. (2025). HyperSight CBCT image quality and metal artifact reduction for adaptive head and neck radiotherapy: Results from a prospective clinical trial. J. Appl. Clin. Med. Phys..

[22] Kunnen B., van de Schoot A.J., Fremeijer K.P., Nicolai-Koornneef E.M., Offereins-van Harten K., Sluijter J.H., Sijtsema N.D., Oomen-de Hoop E., el Yaakoubi A., Froklage F.E. (2024). The added value of a new high-performance ring-gantry CBCT imaging system for prostate cancer patients. Radiother. Oncol..

[23] Schmidt R., Nguyen T., Bicu A.S., Cvachovec P., Siefert V., Eckl M., Willam M., Froelich M.F., Schoenberg S.O., Ehmann M. (2025). Advanced HyperSight imaging for patients with adaptive SBRT of prostate cancer: A longitudinal analysis of tissue demarcation. Radiat. Oncol..

[24] Koo K., Redler G., Semenenko V., Rosenberg S.A., Keit E., Andreozzi J.M. (2025). Localization accuracy of 6-second CBCT for lung IGRT with various breathing patterns. J. Appl. Clin. Med. Phys..

[25] Wessels C., Strzelecki A., Plamondon M., Lehmann M., Peterlik I., Paysan P., Nagy B., Heinz A., Seghers D., Thompson S. (2024). Technical note: Phantom-based evaluation of CBCT dose calculation accuracy for use in adaptive radiotherapy. Med. Phys..

[26] Nelson N., Oare C., Nelson G., Martin T., Huang J., Zhao H. (2025). Feasibility of HyperSight CBCT for adaptive radiation therapy: A phantom benchmark study of dose calculation accuracy and delivery verification on the Halcyon. J. Appl. Clin. Med. Phys..

[27] Martin P.R., Cherpak A., MacDonald R.L., Yashayaeva A., McAloney D., McMaster N., Zhan K., Cwajna S., Patil N., Dahn H.M. (2025). Dose calculation accuracy of clinical radiotherapy plans using next generation cone beam computed tomography imaging technology. Phys. Imaging Radiat. Oncol..

[28] Sijtsema N.D., Penninkhof J.J., van de Schoot A.J., Kunnen B., Sluijter J.H., van de Pol M., Froklage F.E., Dirkx M.L.P., Petit S.F. (2025). Dose calculation accuracy of a new high-performance ring-gantry CBCT imaging system for prostate and lung cancer patients. Radiother. Oncol..

[29] Dusi F., Busato F., Testolin A., Fiorentin D., Tessari F., Nicoletto M., Fusella M. (2025). Evaluation of the dosimetric accuracy of HyperSight CBCT for CBCT-only adaptive radiotherapy workflow. Phys. Med..

[30] Duan J., Pogue J.A., Stanley D.N., Shen S., Viscariello N.N., Cardenas C.E., Popple R.A., Harms J. (2025). Assessing HyperSight iterative CBCT for dose calculation in online adaptive radiotherapy for pelvis and breast patients compared to synthetic CT. J. Appl. Clin. Med. Phys..

[31] Cvachovec P., Bicu A.S., Schmidt R., Siefert V., Eckl M., Willam M., Clausen S., Froelich M.F., Schoenberg S.O., Ehmann M. (2025). Longitudinal stability of HyperSight-CBCT based radiomic features in patients with CT guided adaptive SBRT for prostate cancer. Sci. Rep..

[32] Kubiatowicz A., Sherer M., Owaga A., Sharabi A., Ray X. (2025). Assessing dosimetric benefit from daily online adaptive radiation therapy for esophageal cancer. J. Appl. Clin. Med. Phys..

[33] Bachmann N., Schmidhalter D., Corminboeuf F., Berger M.D., Borbély Y., Ermiş E., Stutz E., Shrestha B.K., Aebersold D.M., Manser P. (2024). Cone beam computed tomography-based online adaptive radiation therapy of esophageal cancer: First clinical experience and dosimetric benefits. Adv. Radiat. Oncol..

[34] Bachmann N., Ahmadsei M., Hürlimann M., Gabrys H.S., Schmidhalter D., Bertholet J., Berger M.D., Borbély Y., Ermiş E., Stutz E. (2025). Cone-beam computed tomography-based online adaptive radiotherapy of esophageal cancer in the neoadjuvant setting: Dosimetric analysis, toxicity and treatment response. Radiother. Oncol..

[35] Galand A., Prunaretty J., Mir N., Morel A., Bourgier C., Aillères N., Azria D., Fenoglietto P. (2023). Feasibility study of adaptive radiotherapy with Ethos for breast cancer. Front. Oncol..

[36] Pogue J.A., Cardenas C.E., Stanley D.N., Stanley C., Hotsinpiller W., Veale C., Soike M.H., Popple R.A., Boggs D.H., Harms J. (2023). Improved dosimetry and plan quality for accelerated partial breast irradiation using online adaptive radiation therapy: A single institutional study. Adv. Radiat. Oncol..

[37] Warden C., Steed K., Hotsinpiller W., Pogue J., Harms J., Soike M., Keene K., Dobelbower M., Bredel M., Stanley D. (2026). CBCT-guided online adaptive radiation therapy for accelerated partial breast irradiation: A single-institution experience. Clin. Breast Cancer.

[38] Parsons D., Domal S., Chambers E., Salazar D., Visak J., Arbab M., Holgado F., Li X., Okoro C., Wandrey N. (2025). Feasibility and impact of a radiation therapy technologist-driven workflow for cone beam computed tomography guided partial breast adaptive radiation therapy. Int. J. Radiat. Oncol. Biol. Phys..

[39] Nelissen K.J., Verbakel W.F.A.R., Middelburg-van Rijn J.G., Rijksen B.L.T., Admiraal M.A., Visser J., van der Himst J., Goudschaal K.N., Bucko E., Slotman B.J. (2024). Clinical implementation of cone beam computed tomography-guided online adaptive radiation therapy in whole breast irradiation. Adv. Radiat. Oncol..

[40] Schiff J.P., Laugeman E., Stowe H.B., Zhao X., Hilliard J., Hawk E., Watkins J., Hatscher C., Badiyan S.N., Samson P.P. (2023). Prospective in silico evaluation of cone-beam computed tomography-guided stereotactic adaptive radiation therapy (CT-STAR) for the ablative treatment of ultracentral thoracic disease. Adv. Radiat. Oncol..

[41] Kishi N., Yoneyama M., Inoo H., Inoue M., Iramina H., Nakakura A., Ono T., Hirashima H., Adachi T., Matsushita N. (2024). Protocol of a phase II study to evaluate the efficacy and safety of deep-inspiration breath-hold daily online adaptive radiotherapy for centrally located lung tumours (PUDDING study). Radiat. Oncol..

[42] Hoppen L., Sarria G.R., Kwok C.S., Boda-Heggemann J., Buergy D., Ehmann M., Giordano F.A., Fleckenstein J. (2023). Dosimetric benefits of adaptive radiation therapy for patients with stage III non-small cell lung cancer. Radiat. Oncol..

[43] Duan J., Harms J., Boggs D.H., Kole A.J., Popple R.A., Stanley D.N., Soike M.H., Viscariello N.N., Cardan R., Cardenas C.E. (2025). Assessing dosimetric benefits of cone beam computed tomography-guided online adaptive radiation treatment frequencies for lung cancer. Adv. Radiat. Oncol..

[44] Li R., Zhuang T., Montalvo S., Wang K., Parsons D., Zhang Y., Iyengar P., Wang J., Godley A., Cai B. (2024). Adapt-on-demand: A novel strategy for personalized adaptive radiation therapy for locally advanced lung cancer. Pract. Radiat. Oncol..

[45] Lukez A., Zhang L., Horwitz E.M., Galloway T.J., Hallman M.A., Wong J.K., Kumar S.S., Shulman R.M., Ma C.C., Eldib A. (2025). Computed tomography-guided online adaptive stereotactic body radiation therapy for liver tumors: A retrospective study. Int. J. Radiat. Oncol. Biol. Phys..

[46] Pierrard J., Deheneffe S., Dechambre D., Sterpin E., Geets X., Van Ooteghem G. (2024). Markerless liver online adaptive stereotactic radiotherapy: Feasibility analysis. Phys. Med. Biol..

[47] Lee A., Pasetsky J., Lavrova E., Wang Y.F., Sedor G., Li F.L., Gallitto M., Garrett M., Elliston C., Price M. (2024). CT-guided online adaptive stereotactic body radiotherapy for pancreas ductal adenocarcinoma: Dosimetric and initial clinical experience. Clin. Transl. Radiat. Oncol..

[48] Jiang D., Peng J., Xu H., Wang D., Xie C., Wang X., Zhou F., Liu H. (2024). Online adaptive radiotherapy in stereotactic body radiotherapy for pancreatic cancer patients. Sci. Rep..

[49] Ogawa A., Nakamura M., Iramina H., Yoshimura M., Mizowaki T. (2023). Potential utility of cone-beam CT-guided adaptive radiotherapy under end-exhalation breath-hold conditions for pancreatic cancer. J. Appl. Clin. Med. Phys..

[50] Rhee D.J., Beddar S., Jaoude J.A., Sawakuchi G., Martin R., Perles L., Yu C., He Y., Court L.E., Ludmir E.B. (2023). Dose escalation for pancreas SBRT: Potential and limitations of using daily online adaptive radiation therapy and an iterative isotoxicity automated planning approach. Adv. Radiat. Oncol..

[51] Takaki M., Hirose T.A., Yoshitake T., Matsumoto K., Shirakawa Y., Wakiyama H., Hisano O., Imafuku H., Ishigami K. (2025). Dosimetric evaluation of cone beam computed tomography-guided online adaptive radiotherapy in gastric mucosa-associated lymphoid tissue lymphoma. Tech. Innov. Patient Support Radiat. Oncol..

[52] Hirose T.A., Takaki M., Shibayama Y., Fukunaga J.I., Kato T., Yoshitake T., Ishigami K. (2024). Evaluation of PTV margin in CBCT-based online adaptive radiation therapy for gastric mucosa-associated lymphoid tissue lymphoma. J. Radiat. Res..

[53] Shibayama Y., Arimura H., Hirose T.A., Takaki M., Fukunaga J.I., Yoshitake T., Kato T., Ishigami K. (2025). Can online adaptive radiation therapy eliminate intrafractional deformation in gastric mucosa-associated lymphoid tissue lymphoma?. Pract. Radiat. Oncol..

[54] Schiff J.P., Stowe H.B., Price A., Laugeman E., Hatscher C., Hugo G.D., Badiyan S.N., Kim H., Robinson C.G., Henke L.E. (2022). In silico trial of computed tomography-guided stereotactic adaptive radiation therapy (CT-STAR) for the treatment of abdominal oligometastases. Int. J. Radiat. Oncol. Biol. Phys..

[55] van Lieshout E., van Werkhoven L.A., Hoogeman M.S., Nout R.A., Milder M.T.W., Nuyttens J. (2025). Reducing number of treatment fractions for patients with abdominal lymph node oligometastases: The need for online adaptive radiation therapy to provide personalized adaptive fractionation. Int. J. Radiat. Oncol. Biol. Phys..

[56] Wurschi G., Voigt A., Murr N., Riede C., Schwedas M., Romer M., Drozdz S., Pietschmann K. (2025). CBCT-based online adaptive, ultra-hypofractionated radiotherapy for prostate cancer: First clinical experiences. Medicina.

[57] Zhu L.L., Bredfeldt J.S., Hu Y.H., Hancox C., Guthier C.V., Quirk S., Pursley J., Kamran S.C., McClatchy D., Nguyen P.L. (2025). CT online adaptive radiotherapy is associated with dosimetric and acute toxicity improvements in prostate cancer treatment. Radiother. Oncol..

[58] Khouya A., Pottgen C., Hoffmann C., Ringbaek T.P., Lubcke W., Indenkampen F., Guberina M., Guberina N., Gauler T., Stuschke M. (2023). Adaptation Time as a Determinant of the dosimetric effectiveness of online adaptive radiotherapy for bladder cancer. Cancers.

[59] Waters M., Price A., Laugeman E., Henke L., Hugo G., Stowe H., Andruska N., Brenneman R., Hao Y., Green O. (2024). CT-based online adaptive radiotherapy improves target coverage and organ at risk (OAR) avoidance in stereotactic body radiation therapy (SBRT) for prostate cancer. Clin. Transl. Radiat. Oncol..

[60] Pottgen C., Hoffmann C., Gauler T., Guberina M., Guberina N., Ringbaek T., Santiago Garcia A., Krafft U., Hadaschik B., Khouya A. (2023). Fractionation versus adaptation for compensation of target volume changes during online adaptive radiotherapy for bladder cancer: Answers from a prospective registry. Cancers.

[61] Sun S., Gong X., Liang Y., Sun Y., Que D., Xie Y., He S., He L., Liang H., Wang Y. (2025). Evaluating the implementation of fan-beam CT-guided online adaptive re-planning in definitive cervical cancer radiotherapy. Front Oncol..

[62] Wang G., Chen Y., Wang Z., Zeng Z., Sun Y., Zhou B., Yang B., Qiu J., Yan J., Hu K. (2025). A prospective single-arm study of daily online adaptive radiation therapy for cervical cancer with reduced planning target volume margin: Acute toxicity and dosimetric outcomes. Int. J. Radiat. Oncol. Biol. Phys..

[63] Jiang D., Yang C., Sun S., Wang D., Xiao Z., Hu J., Mei Z., Xie C., Liu H., Qiu H. (2024). First implementation and results of online adaptive radiotherapy for cervical cancer based on CT-Linac combination. Front. Oncol..

[64] Wang G., Wang Z., Zhang Y., Sun X., Sun Y., Guo Y., Zeng Z., Zhou B., Hu K., Qiu J. (2024). Daily online adaptive radiation therapy of postoperative endometrial and cervical cancer with PTV margin reduction to 5 mm: Dosimetric outcomes, acute toxicity, and first clinical experience. Adv. Radiat. Oncol..

[65] Wang G., Wang Z., Guo Y., Zhang Y., Qiu J., Hu K., Li J., Yan J., Zhang F. (2024). Evaluation of PTV margins with daily iterative online adaptive radiotherapy for postoperative treatment of endometrial and cervical cancer: A prospective single-arm phase 2 study. Radiat. Oncol..

[66] Bak M.E., Jensen N.K.G., Nottrup T.J., Mathiesen H.F., Roed H., Sjolin M., Kjaer-Kristoffersen F., Hansen V.N., Vogelius I.R. (2023). Clinical experiences with online adaptive radiotherapy of vulvar carcinoma. Acta. Oncol..

[67] Guberina M., Santiago Garcia A., Khouya A., Pottgen C., Holubyev K., Ringbaek T.P., Lachmuth M., Alberti Y., Hoffmann C., Hlouschek J. (2023). Comparison of online-onboard adaptive intensity-modulated radiation therapy or volumetric-modulated arc radiotherapy with image-guided radiotherapy for patients with gynecologic tumors in dependence on fractionation and the planning target volume margin. JAMA. Netw. Open.

[68] Malygina H., Salazar Zuniga B., Auerbach H., Ries M., Dzierma Y., Hecht M., Palm J. (2025). Daily online adaptation enhances target coverage in prostate cancer radiotherapy: A retrospective analysis. Front. Oncol..

[69] Sa Y., Basith A., Apsani R.C., Gurusamy V.M., Khemissi A., Paloor S., Divakar S., McCabe S., Hammoud R., Al-Hammadi N. (2025). Evaluating intra-fraction motion and its dosimetric impact in ethos online adaptive radiotherapy for prostate cancer. Precis Radiat. Oncol..

[70] Preziosi F., Boschetti A., Catucci F., Votta C., Vellini L., Menna S., Quaranta F., Pilloni E., D’Aviero A., Aquilano M. (2025). AI-driven online adaptive radiotherapy in prostate cancer treatment: Considerations on activity time and dosimetric benefits. Radiat. Oncol..

[71] Malygina H., Auerbach H., Ries M., Nuesken F., Salazar Zuniga B., Moumeniahangar S., Oeschger F., Hecht M., Palm J., Dzierma Y. (2025). Intra-adaptational changes in online adaptive radiotherapy: From the ideal to the real dose. Strahlenther. Onkol..

[72] Fischer J., Fischer L.A., Bensberg J., Bojko N., Bouabdallaoui M., Frohn J., Huttenrauch P., Tegeler K., Wagner D., Wenzel A. (2025). CBCT-based online adaptive radiotherapy of the prostate bed: First clinical experience and comparison to nonadaptive conventional IGRT. Strahlenther. Onkol..

[73] Byrne M., Teh A.Y.M., Archibald-Heeren B., Hu Y., Rijken J., Luo S., Aland T., Greer P. (2024). Intrafraction motion and margin assessment for Ethos online adaptive radiotherapy treatments of the prostate and seminal vesicles. Adv. Radiat. Oncol..

[74] Brennsaeter J.A., Dahle T.J., Moi J.N., Svanberg I.F., Haaland G.S., Pilskog S. (2023). Reduction of PTV margins for elective pelvic lymph nodes in online adaptive radiotherapy of prostate cancer patients. Acta Oncol..

[75] Zwart L.G.M., Ong F., Ten Asbroek L.A., van Dieren E.B., Koch S.A., Bhawanie A., de Wit E., Dasselaar J.J. (2022). Cone-beam computed tomography-guided online adaptive radiotherapy is feasible for prostate cancer patients. Phys. Imaging Radiat. Oncol..

[76] Morgan H.E., Wang K., Yan Y., Desai N., Hannan R., Chambers E., Cai B., Lin M.H., Sher D.J., Wang J. (2023). Preliminary evaluation of PTV margins for online adaptive radiation therapy of the prostatic fossa. Pract. Radiat. Oncol..

[77] Langen K.M., Willoughby T.R., Meeks S.L., Santhanam A., Cunningham A., Levine L., Kupelian P.A. (2008). Observations on real-time prostate gland motion using electromagnetic tracking. Int. J. Radiat. Oncol. Biol. Phys..

[78] Fischer J., Fischer L.A., Bensberg J., Bojko N., Bouabdallaoui M., Frohn J., Huttenrauch P., Klingebiel M., Schmitt D., Tegeler K. (2025). CBCT-based online adaptive radiotherapy of the bladder—Geometrical and dosimetrical considerations compared to conventional IGRT. Radiat. Oncol..

[79] Varga L., Galdi A., Szegedi D., Herein A., Pulugor D., Nahaji I., Gesztesi L., Jorgo K., Takacsi Nagy Z., Polgar C. (2025). Reduction of the planning target volume with daily online adaptive radiotherapy in bladder cancer. Strahlenther. Onkol..

[80] Azzarouali S., Goudschaal K., Visser J., Hulshof M., Admiraal M., van Wieringen N., Nieuwenhuijzen J., Wiersma J., Daniels L., den Boer D. (2023). Online adaptive radiotherapy for bladder cancer using a simultaneous integrated boost and fiducial markers. Radiat. Oncol..

[81] Astrom L.M., Behrens C.P., Calmels L., Sjostrom D., Geertsen P., Mouritsen L.S., Serup-Hansen E., Lindberg H., Sibolt P. (2022). Online adaptive radiotherapy of urinary bladder cancer with full re-optimization to the anatomy of the day: Initial experience and dosimetric benefits. Radiother. Oncol..

[82] VanderWalde N.A., Chi M.T., Hurria A., Galsky M.D., Nielsen M.E. (2016). Treatment of muscle invasive bladder cancer in the elderly: Navigating the trade-offs of risk and benefit. World J. Urol..

[83] Gupta S., Hensley P.J., Li R., Choudhury A., Daneshmand S., Faltas B.M., Flaig T.W., Grass G.D., Grivas P., Hansel D.E. (2026). Bladder preservation strategies in muscle-invasive bladder cancer: Recommendations from the international bladder cancer group. Eur. Urol..

[84] Maebayashi T., Ishikawa H., Yorozu A., Yoshida D., Katoh H., Nemoto K., Ishihara S., Takemoto S., Ishibashi N., Tokumaru S. (2014). Patterns of practice in the radiation therapy for bladder cancer: Survey of the Japanese Radiation Oncology Study Group (JROSG). Jpn. J. Clin. Oncol..

[85] Aizawa R., Sakamoto M., Orito N., Kono M., Ogura M., Negoro Y., Sagoh T., Tsukahara K., Komatsu K., Noguchi M. (2017). The use of external-beam radiotherapy for muscle-invasive bladder cancer in elderly or medically-fragile patients. Anticancer Res..

[86] Tokuda P.J.K., Aizawa R., Iramina H., Ogata T., Hirashima H., Kita Y., Sumiyoshi T., Kobayashi T., Mizowaki T. (2026). Online adaptive radiation therapy for muscle-invasive bladder cancer: Short-course and high-precision definitive treatment for elderly or medically fragile patients. Jpn. J. Radiol..

[87] Yen A., Zhong X., Lin M.H., Nwachukwu C., Albuquerque K., Hrycushko B. (2024). Improved dosimetry with daily online adaptive radiotherapy for cervical cancer: Waltzing the pear. Clin. Oncol..

[88] Peng H., Zhang J., Xu N., Zhou Y., Tan H., Ren T. (2023). Fan beam CT-guided online adaptive external radiotherapy of uterine cervical cancer: A dosimetric evaluation. BMC Cancer.

[89] Yen A., Zhong X., Lin M.H., Nwachukwu C., Albuquerque K., Hrycushko B. (2024). Optimizing online adaptation timing in the treatment of locally advanced cervical cancer. Pract. Radiat. Oncol..

[90] Ghimire R., Moore K.L., Branco D., Rash D.L., Mayadev J., Ray X. (2023). Forecasting patient-specific dosimetric benefit from daily online adaptive radiotherapy for cervical cancer. Biomed. Phys. Eng. Express.

[91] Yen A., Choi B., Inam E., Yeh A., Lin M.H., Park C., Hrycushko B., Nwachukwu C., Albuquerque K. (2023). Spare the bowel, don’t spoil the target: Optimal margin assessment for online cone beam adaptive radiation therapy (OnC-ART) of the cervix. Pract. Radiat. Oncol..

[92] Zeng Z., Chen Y., Qiu J., Yang B., Wang Z., Meng X., Sun Y., Yan J., Hu K., Zhang F. (2025). Moderately hypofractionated online adaptive radiotherapy (SWIFT-1) in cervical cancer patients: Study protocol for a multi-centered, open-label, two-arm, phase III, randomized controlled study. Radiat. Oncol..

[93] Pierrard J., Dechambre D., Ooteghem G.V. (2025). Investigation of changes in planning target volume and regression probability of rectal boost using in-silico cone-beam computed tomography-guided online-adaptive radiotherapy. Phys. Imaging Radiat. Oncol..

[94] Storm K.S., Astrom L.M., Sibolt P., Behrens C.P., Persson G.F., Serup-Hansen E. (2024). ROAR-A: Re-optimization based online adaptive radiotherapy of anal cancer, a prospective phase II trial protocol. BMC Cancer.

[95] Astrom L.M., Behrens C.P., Storm K.S., Sibolt P., Serup-Hansen E. (2022). Online adaptive radiotherapy of anal cancer: Normal tissue sparing, target propagation methods, and first clinical experience. Radiother. Oncol..

[96] de Jong R., Crama K.F., Visser J., van Wieringen N., Wiersma J., Geijsen E.D., Bel A. (2020). Online adaptive radiotherapy compared to plan selection for rectal cancer: Quantifying the benefit. Radiat. Oncol..

[97] Sher D.J., Avkshtol V., Lin M.H., Wang J., Chen L., Liao C.Y., Hughes R., Domal S., Ahn C., Moon D.H. (2025). Impact of daily adaptive head and neck radiotherapy on toxicity and quality-of-life: Results of the DARTBOARD phase II randomized trial. J. Natl. Cancer Inst..

[98] Liu W., Schiff J.P., Hassanzadeh C., Miller K., Hatscher C., Beckert R., Price A., Daly M., Brenneman R., Henke L. (2025). Palliative expeditiously adaptive quad shot radiotherapy for head and neck cancers (PEAQ-RT). Clin. Transl. Radiat. Oncol..

[99] Blumenfeld P., Arbit E., Den R., Salhab A., Michaeli T.F., Wygoda M., Hillman Y., Pfeffer R.M., Fang M., Misrati Y. (2024). Real world clinical experience using daily intelligence-assisted online adaptive radiotherapy for head and neck cancer. Radiat. Oncol..

[100] Dohopolski M., Visak J., Choi B., Meng B., Parsons D., Zhong X., Inam E., Avkshtol V., Moon D., Sher D. (2024). In silico evaluation and feasibility of near margin-less head and neck daily adaptive radiotherapy. Radiother. Oncol..

[101] Guberina M., Guberina N., Hoffmann C., Gogishvili A., Freisleben F., Herz A., Hlouschek J., Gauler T., Lang S., Stähr K. (2024). Prospects for online adaptive radiation therapy (ART) for head and neck cancer. Radiat. Oncol..

[102] Avkshtol V., Meng B., Shen C., Choi B.S., Okoroafor C., Moon D., Sher D., Lin M.H. (2023). Early experience of online adaptive radiation therapy for definitive radiation of patients with head and neck cancer. Adv. Radiat. Oncol..

[103] Barragán-Montero A.M., Van Ooteghem G., Dumont D., Rivas S.T., Sterpin E., Geets X. (2023). Dosimetrically triggered adaptive radiotherapy for head and neck cancer: Considerations for the implementation of clinical protocols. J. Appl. Clin. Med. Phys..

[104] Cushman T.R., Shirvani S., Khan M., Kumar R. (2018). The effect of early versus delayed radiation therapy on length of hospital stay in the palliative setting. Ann. Palliat. Med..

[105] Nelissen K.J., Versteijne E., Senan S., Hoffmans D., Slotman B.J., Verbakel W.F.A.R. (2023). Evaluation of a workflow for cone-beam CT-guided online adaptive palliative radiotherapy planned using diagnostic CT scans. J. Appl. Clin. Med. Phys..

[106] Nelissen K.J., Versteijne E., Senan S., Rijksen B., Admiraal M., Visser J., Barink S., de la Fuente A.L., Hoffmans D., Slotman B.J. (2023). Same-day adaptive palliative radiotherapy without prior CT simulation: Early outcomes in the FAST-METS study. Radiother. Oncol..

[107] Kejda A., Quinn A., Wong S., Lowe T., Fent I., Gargett M., Roderick S., Grimberg K., Bergamin S., Eade T. (2023). Evaluation of the clinical feasibility of cone-beam computed tomography guided online adaption for simulation-free palliative radiotherapy. Phys. Imaging Radiat. Oncol..

[108] Schuurhuizen C.S.E.W., Milder M.T.W., Sluijter J.H., Dirkx M.L.P., Nuyttens J.J.M.E. (2025). Clinical feasibility of treatment planning on a diagnostic CT scan without or with single fraction plan adaptation in patients with stage II/III rectal cancer. Radiother. Oncol..

[109] Price A.T., Schiff J.P., Silberstein A., Beckert R., Zhao T., Hugo G.D., Samson P.P., Laugeman E., Henke L.E. (2024). Feasibility of simulation free abdominal stereotactic adaptive radiotherapy using an expedited pre-plan workflow. Phys. Imaging Radiat. Oncol..

[110] MacDonald R.L., Fallone C., Chytyk-Praznik K., Robar J., Cherpak A. (2024). The feasibility of CT simulation-free adaptive radiation therapy. J. Appl. Clin. Med. Phys..

[111] McGrath K.M., MacDonald R.L., Robar J.L., Cherpak A. (2025). Direct-to-treatment adaptive radiation therapy: Live planning of spine metastases using novel cone beam computed tomography. Int. J. Radiat. Oncol. Biol. Phys..

[112] Wang D., Kim H., Zhuang T., Visak J.D., Cai B., Parsons D.D.M., Jiang S., Godley A.R., Lin M.H. (2025). Simulation-omitting and using library patients for pre-planning online adaptive radiotherapy (SUPPORT): A feasibility study for spine stereotactic ablative radiotherapy (SAbR) patients. Cancers.

[113] Shelley C., Hollingdale R., Bolt M., Rashid M., Reinlo S., Fazel N., Thorpe C., Chadwick S., Barnard A., South C.P. (2022). Radiographer-led, CBCT-guided online adaptive radiotherapy for muscle invasive bladder cancer using artificial intelligence. Int. J. Radiat. Oncol. Biol. Phys..

[114] Visak J., Washington B., Liao C.Y., Domal S., Parsons D., Zhang Y., Badiyan S., Westover K., Lin M.H. (2025). Improving efficiency in lung SAbR planning using integrated tools for X-ray based adaptive radiotherapy. J. Appl. Clin. Med. Phys..

[115] Yashayaeva A., MacDonald R.L., Cherpak A. (2025). Body contour adaptation for weight-loss and bolus for head and neck radiotherapy on Ethos version 2.0 and HyperSight: Synthetic CT versus direct calculation. J. Appl. Clin. Med. Phys..

[116] Page M.J., McKenzie J.E., Bossuyt P.M., Boutron I., Hoffmann T.C., Mulrow C.D., Shamseer L., Tetzlaff J.M., Akl E.A., Brennan S.E. (2021). The PRISMA 2020 statement: An updated guideline for reporting systematic reviews. BMJ.

